# Use of Tocilizumab Followed by Rituximab Desensitization on Relapsing Myelin Oligodendrocyte Antibody Disease: A Case Report

**DOI:** 10.7759/cureus.52374

**Published:** 2024-01-16

**Authors:** Kelsey C Dayrit, Evelyn O Chua-Ley

**Affiliations:** 1 Section of Neurology, Cardinal Santos Medical Center, San Juan, PHL; 2 Department of Clinical Neurosciences, University of the East Ramon Magsaysay Memorial Medical Center, Quezon, PHL

**Keywords:** demyelinating dieases, rituximab desensitization, rituximab, tocilizumab, mog associated antibody disease (mogad)

## Abstract

Myelin oligodendrocyte antibody disease is a demyelinating disorder that usually presents with a monophasic course. Relapse in this demyelinating disorder is rare, and those who relapse have a weaker response to standard therapy. In this case report, we report a three-year follow-up on a case of a female patient who was diagnosed with myelin oligodendrocyte antibody disease at 21 years old. The patient initially presented with transverse myelitis followed by optic neuritis five months after the onset of transverse myelitis. On relapse, the patient was initially treated with rituximab only to present with type 1 hypersensitivity reaction. Due to the hypersensitivity reaction, the treatment regimen was shifted to tocilizumab, for which she completed a total of five cycles. With tocilizumab treatment, she was noted to have one relapse of symptoms triggered by COVID-19 infection. However, due to tocilizumab-associated alopecia, the patient was shifted to rituximab infusion with desensitization. She then underwent four cycles of rituximab with desensitization, which she tolerated well, and is now in full remission after the fourth cycle of rituximab with no residual deficits.

As relapse in myelin oligodendrocyte antibody disease is rare, studies regarding the use of tocilizumab and rituximab as second-line treatment for this disorder are limited. Literature regarding treatment with rituximab infusion with desensitization is even more limited. This case report highlights the potential use of tocilizumab and rituximab in relapsing cases of myelin oligodendrocyte antibody disease, as well as the need for additional literature regarding the use of tocilizumab and rituximab with or without desensitization in relapse in myelin oligodendrocyte antibody disease.

## Introduction

Myelin oligodendrocyte IgG is a type of protein found on the surface of oligodendrocytes. It was previously associated with multiple sclerosis but has since been recognized as a separate demyelinating disease covering a wide spectrum of manifestations and is defined by seropositivity to myelin oligodendrocyte IgG antibody by use of radioimmunoassay technique that could detect self-antigen tetramers. Currently, live cell-based assays are the gold standard for myelin oligodendrocyte IgG antibody testing with 98.1-100% specificity and 23.1-27.1% sensitivity. Myelin oligodendrocyte antibody disease (MOGAD) is an immune-mediated neurologic disorder resulting in demyelination, mainly targeting the spinal cord, optic nerves, and the brain. Clinical manifestations of patients presenting with MOGAD vary in the literature; however, attacks were reported to commonly present as optic neuritis or transverse myelitis that can be seen either alone or in combination. Acute disseminated encephalomyelitis (ADEM) can also be one of the possible presentations of MOGAD. The frequency of this is variable but was most commonly reported as an initial presentation in pediatric cases of MOGAD. MOGAD can be differentiated from multiple sclerosis and neuromyelitis optica spectrum disorder (NMOSD) by its differences in clinical as well as radiologic presentation. It can also be differentiated through cerebrospinal fluid analysis. The incidence and prevalence of MOGAD is largely unverified. Sources have suggested that the incidence of MOGAD can range from 1.6 to 3.4 per 1,000,000 persons per year. It was also noted that half of the reported cases of MOGAD were from the pediatric population [1]. No major sex difference has been found with the median age of onset at 20-30 years [2,3].

The course of MOGAD can either be monophasic (single attack) or relapsing. In acute attacks, the treatment of choice is high-dose intravenous steroid therapy followed by slow tapering of steroids. Failure of initial acute therapy or patient refractory to initial acute therapy may undergo plasma exchange or intravenous immunoglobulin (IVIG) infusion. On the other hand, relapsing MOGAD may be treated with other disease-modifying drugs like rituximab, tocilizumab, mycophenolate mofetil, azathioprine, or intermittent IVIG infusion. Here, we present a relapsing case of MOGAD treated with tocilizumab followed by rituximab infusion with desensitization.

## Case presentation

**Figure 1:**
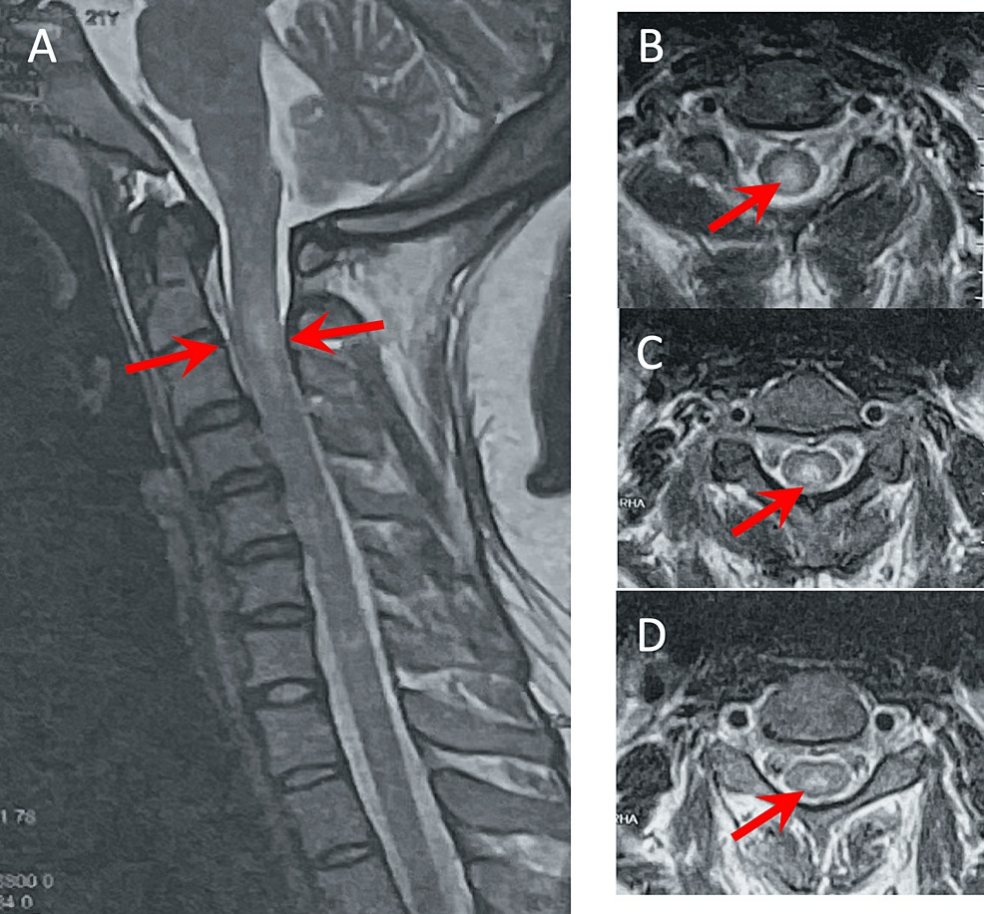
T2 weighted cervical MRI study showing focal 2.0 cm heterogeneously enhancing lesion (red arrows) involving the central intramedullary region at C2 to upper C3 levels, associated with subtle cord expansion and minimal adjacent cord edema A: T2 weighted cervicothoracic MRI sagittal cut B: T2 weighted cervical MRI transverse cut at upper C2 C: T2 weighted cervical MRI transverse cut at lower C2 D: T2 weighted cervical MRI transverse cut at upper C3

**Figure 2:**
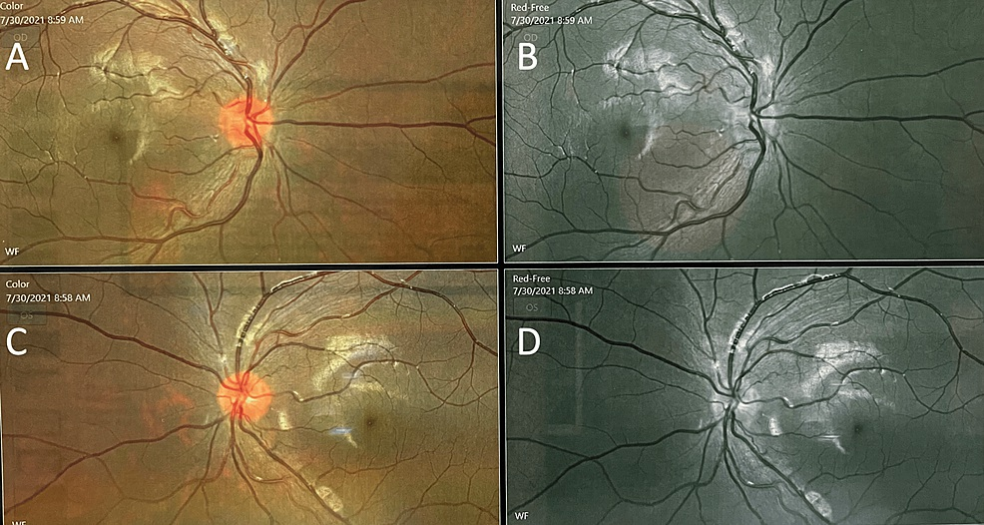
Optical coherence tomography showing average retinal nerve fibers and normal ganglion cell analysis on both eyes A-B: right eye; C-D: left eye

A 21-year-old right-handed female presented with a history of a cape-like distribution of numbness over the upper back and down to the posterolateral upper extremities at the level of C4-C8 and a band-like sensation over T5-T11. Numbness during this time was described as decreased sensation to crude touch and pain over the mentioned regions, with paresthesia most prominent over the upper extremities described as a tingling sensation. No associated motor deficit, blurring of vision, cognitive decline, headache, dizziness, nausea or vomiting, or weight loss/gain were noted. Due to the persistence of symptoms, the patient sought multiple consults. A cervicothoracic, shown in Figure 1, and cranial MRI were done, revealing a heterogeneously enhancing lesion involving the central intramedullary region at C2 to upper C3 levels associated with subtle cord expansion and minimal adjacent cord edema. A cranial MRI was also requested at the time, which was noted to be unremarkable. On further review of history, symptoms were noted to be preceded by COVID-19 infection a month prior to the onset of symptoms. She was then initially treated as a case of COVID-associated transverse myelitis vs clinically isolated syndrome (CIS) and was given pulse therapy with methylprednisolone 1 g IV Q24 for a total of five. Baseline optical coherence tomography, shown in Figure 2, was also done during the first admission, revealing a normal ganglion cell analysis of both eyes. In the interim, the patient's symptoms were noted to minimally improve with rehabilitation but with no progression of symptoms.

**Figure 3:**
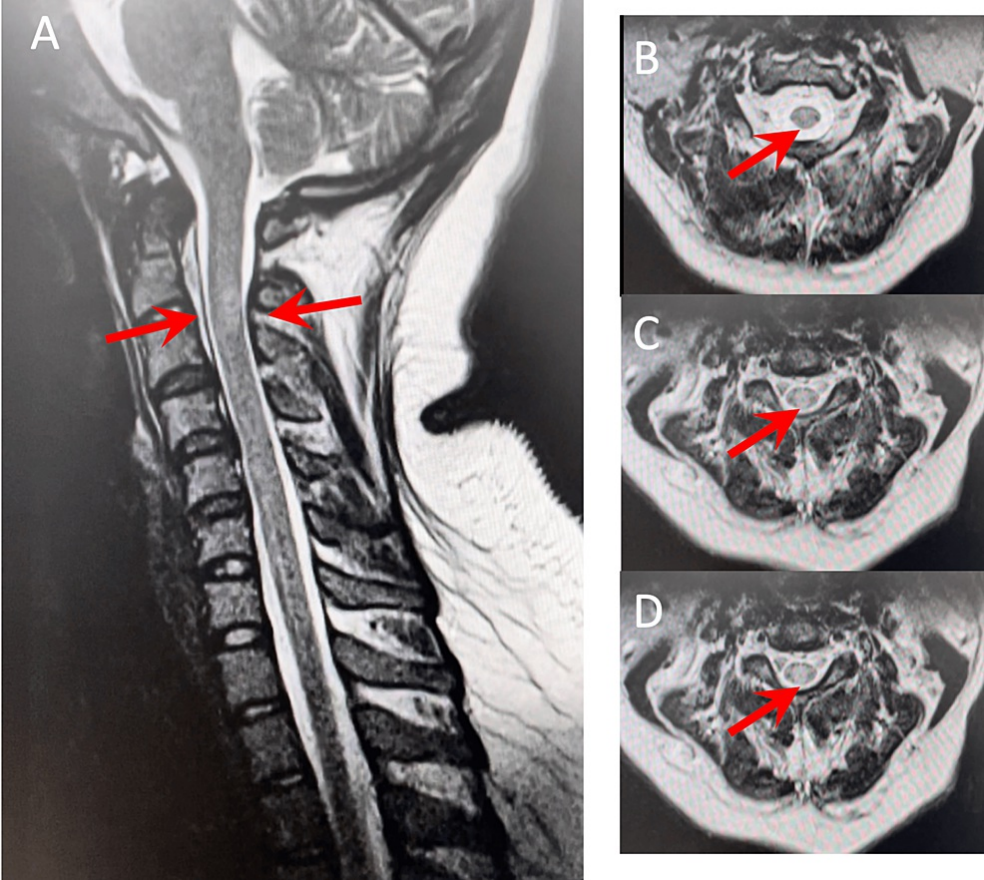
T2 weighted cervical MRI showing focal ovoid signal abnormality involving the dorsal aspect of the C2-3 level of the upper cervical spinal cord A: T2 weighted cervicothoracic MRI sagittal cut B: T2 weighted cervical MRI transverse cut at upper C2 C: T2 weighted cervical MRI transverse cut at lower C2 D: T2 weighted cervical MRI transverse cut at upper C3

Five months after the initial diagnosis of COVID-associated transverse myelitis vs CIS, the patient now presented with complaints of blurring of vision over the right eye associated with pain on eye movement. A repeat craniocervical MRI shown in Figure 3 was done, revealing the previously noted focal ovoid non-enhancing signal abnormality involving the dorsal aspect of the C2-3 level of the upper cervical spinal cord with no accompanying cord expansion. The rest of the visualized cervical spinal cord demonstrated no other additional intrinsic signal abnormality. Visual evoked potential (VEP) was also done, revealing an abnormal VEP study, showing conduction defect/block with stimulation of the right eye. 

During this time, she underwent another pulse therapy with methylprednisolone 1 g IV Q24 for a total of five days. Serum myelin oligodendrocyte glycoprotein (MOG)** **IgG and aquaporin-4 antibody were requested. MOG IgG result was noted to be positive, while the aquaporin-4 antibody was noted to be negative. She was then treated as a case of MOG-associated antibody disease (MOGAD). Rituximab infusion was done a month after the onset of optic neuritis; however, the patient was noted to have a type 1 hypersensitivity reaction presenting with angioedema and maculopapular rashes. A monthly infusion with tocilizumab 320 mg IV to run for two hours was then initiated as an alternative, which she underwent for a total of five cycles. With tocilizumab infusion, the patient was noted to only have one relapse during her fourth cycle due to another COVID-19 infection. During tocilizumab treatment, the patient did not have any hypersensitivity reaction but was noted to have tocilizumab-associated alopecia. Due to this tocilizumab-associated side effect, the patient requested for a change in treatment; hence, the patient underwent rituximab desensitization. The summary of treatment is shown inTable 1.

**Table 1:** Treatment summary

Treatment	Date given	Completed or aborted
Methylprednisolone 1 g given intravenously every 24 hours for five days	July 2021	Completed
Methylprednisolone 1 g given intravenously every 24 hours for five days	December 2021	Completed
Rituximab 500 mg given via intravenous infusion	January 2022	Aborted due to type 1 hypersensitivity reaction
Tocilizumab 320 mg given via intravenous infusion	March 2022	Completed
Tocilizumab 320 mg given via intravenous infusion	April 2022	Completed
Tocilizumab 320 mg given via intravenous infusion	May 2022	Completed
Tocilizumab 320 mg given via intravenous infusion	June 2022	Completed
Tocilizumab 320 mg given via intravenous infusion	July 2022	Completed
Rituximab 500 mg given via intravenous infusion with desensitization	August 2022	Completed
Rituximab 500 mg given via intravenous infusion with desensitization	September 2022	Aborted due to type 1 hypersensitivity reaction
Rituximab 500 mg given via intravenous infusion with desensitization	October 2022	Completed
Rituximab 500 mg given via intravenous infusion with desensitization	March 2023	Completed
Rituximab 500 mg given via intravenous infusion with desensitization	October 2023	Completed

Desensitization was done by preparing three different solutions containing rituximab at different concentrations, as seen in Table 2. This was then infused, as shown in Table 3. The patient was able to tolerate her first rituximab infusion with desensitization but was noted to have a recurrence of type 1 hypersensitivity reaction on her second session. The second session was then aborted and was delayed for one month. The patient then tolerated the following rituximab infusion and has currently finished her fourth session. 

**Table 2:** Rituximab desensitization dose

3 Solutions	Dilution	Total drug/solution	Volume	Concentration
Solution 1	1/100^th^	5 mg	250 ml	0.02 mg/ml
Solution 2	1/10^th^	50 mg	250 ml	0.2 mg/ml
Solution 3	Standard concentration	500 mg	250 ml	2 mg/ml

**Table 3:** Rituximab desensitization infusion

Step	Solution #	Rate ml/hr	Time (min)	Vol/step	Dose/step	Cumulative dose
1	1	2	15	0.5	0.01	0.010
2		5	15	1.25	0.025	0.035
3		10	15	2.5	0.05	0.085
4		20	15	5	0.1	0.185
5	2	5	15	1.25	0.25	0.435
6		10	15	2.5	0.5	0.935
7		20	15	5	1.0	1.935
8		40	15	10	2.0	3.935
9	3	10	15	2.5	5.0	8.935
10		20	15	5	10.0	18.935
11		30	15	7.5	15.0	33.935
12		40	15	10	20.0	53.935
13		50	15	12.5	25.0	58.935
14		60	210.5325	210.5325	421.065	500.000

## Discussion

Myelin oligodendrocyte antibody disease is an immune-mediated neurologic disorder resulting in demyelination mainly targeting the spinal cord, optic nerves, and the brain. Clinical manifestations of patients presenting with MOGAD vary in the review of literature; however, attacks were reported to commonly present as optic neuritis (unilateral or bilateral) or transverse myelitis that can be seen either alone or in combination. At times, MOGAD can also present as an acute disseminated encephalomyelitis (ADEM), presenting as altered mental status with focal neurologic deficits. ADEM was, however, more commonly reported in pediatric cases [1]. Proposed diagnostic criteria from a single center for MOG antibody-associated disease require the following: 1) serum positivity for MOG IgG by cell-based assay and 2) a clinical presentation consistent with any of the following central nervous system syndromes: ADEM, optic neuritis, transverse myelitis, brain or brainstem demyelinating syndrome or any combination of these with the exclusion of an alternative diagnosis [4-6]. This proposed criterion was mostly used in the diagnosis of MOGAD. However, variation in the diagnostic approaches among different medical institutions still exists as validation of the previously discussed diagnostic criteria is still warranted. Acute attacks of MOGAD were reported to have a tendency to be severe. High-dose glucocorticoid therapy is considered the first-line treatment for acute attacks of MOGAD. Early initiation of treatment during acute attacks was reported to lessen the risk of having residual deficits. Table 4 shows the treatment of choice for MOGAD. 

**Table 4:** Treatment of choice for myelin oligodendrocyte antibody disease

Treatment of acute attacks	Failure of initial acute therapy or refractory to initial acute therapy or incomplete response
High dose intravenous steroid (methylprednisolone) – 1000 mg in adults or 20-30 mg/kg per day in children for five days followed by slow tapering of steroids.	1) Plasma exchange – one exchange every other day for five to seven exchanges; 2) intravenous immunoglobulin – alternative to plasma exchange; total dose: 2 g/kg divided over five days.

As compared to multiple sclerosis and NMOSD, the majority of patients with MOGAD were reported to have a single attack without recurrence. This type of course is called monophasic. However, for patients with relapsing diseases or who are refractory to initial acute therapy, different steroid-sparing treatment modalities are employed [1]. The risk of a relapsing course was noted to increase with the persistence of MOG antibody in some studies, as compared with those with transient seropositivity after treatment [5].

In relapsing MOGAD, different steroid-sparing treatment modalities are available. Rituximab and tocilizumab are one of these steroid-sparing treatment modalities. Rituximab is one of the preferred and most studied treatments for relapsing MOGAD. Rituximab is a monoclonal antibody that targets CD20 in B cells and is commonly used in oncologic and hematologic malignancies. In MOGAD, a large international retrospective cohort study regarding the use of rituximab was done in 2020 with 121 patients. This study showed that in the relapsing group, the overall relapse rate declined by 37% when rituximab was used as the first line and 26% when used after other steroid-sparing immunotherapies [7]. Although rituximab is the commonly preferred treatment for relapsing MOGAD, hypersensitivity reactions with this medication limit its use. Hypersensitivity reaction with rituximab can either be a type 1 immunoglobulin E (IgE)-mediated reaction or a cytokine-mediated reaction. Some have also reported a mixture of both IgE and cytokine-mediated reactions secondary to rituximab use [8]. If treatment with rituximab is warranted with no equally effective alternatives, desensitization may be done in patients presenting with IgE-dependent or IgE-independent hypersensitivity reactions, just like what was done in the patient presented. This is, however, contraindicated in patients with a history of Stevens-Johnson's syndrome, toxic epidermal necrolysis, serum sickness, or hemolytic anemia. In rituximab desensitization, a referral to an allergologist may be done to facilitate rituximab desensitization protocol. A complete allergy history must be taken, and the patient should be admitted to an intensive care setting where there can be 1:1 nursing supervision of the infusion. Rituximab has shown great potential as a treatment for relapsing MOGAD; hence, its use has greatly increased. Due to its popularity and effectiveness, patients with hypersensitivity reaction even undergo desensitization for this steroid-sparing immunotherapy. On the other hand, tocilizumab is an interleukin-6 (IL-6) inhibitor used in both neuromyelitis optica (NMO) and MOGAD. Since both NMO and MOGAD display both antibody and complement-mediated central nervous system (CNS) injury with elevated IL-6, IL-6 blockade was theorized to be beneficial in both diseases. In a retrospective multicenter trial published in 2022, the safety and efficacy of tocilizumab in MOGAD and NMO-SD were studied. In this study, patients received tocilizumab for 23.8 months, with an IV dose of 8.0 mg/kg every 31.6 days. For MOGAD, the median annualized relapse rate (ARR) decreased from 1.75 (range: 0.5-5) to 0 (range: 0-0.9; p=0.0011) under tocilizumab [9]. In patients who are hypersensitive to rituximab and cannot tolerate desensitization, tocilizumab has shown great potential as an alternative steroid-sparing immunotherapy for patients with relapsing MOGAD. 

## Conclusions

Myelin oligodendrocyte antibody disease is an underreported demyelinating disease of the central nervous system. However, due to recent interest in this disease, there has been an increase in the number of studies regarding its treatment. Acute attacks of MOGAD are usually treated with high-dose steroid therapy. However, for those with relapsing disease, steroid-sparing immunotherapies are the treatment of choice. Of these steroid-sparing immunotherapies, rituximab and tocilizumab were one of the most cited treatments. Rituximab was shown to be effective in patients with MOGAD, especially those with relapsing disease. However, hypersensitivity reactions with rituximab were commonly observed, thus limiting the use of rituximab as first-line therapy for many cases. If treatment with rituximab is warranted with no equally effective alternatives, desensitization may be done in patients presenting hypersensitivity reactions. Tocilizumab, on the other hand, is an interleukin-6 inhibitor used in both neuromyelitis optica and MOGAD. For patients who cannot tolerate rituximab and its desensitization, this medication may be a good alternative.
